# Quantum Mechanical Aspects in the Pathophysiology of Neuropathic Pain

**DOI:** 10.3390/brainsci12050658

**Published:** 2022-05-17

**Authors:** Sager Nawafleh, Abdallah Barjas Qaswal, Obada Alali, Fuad Mohammed Zayed, Ahmed Mahmoud Al-Azzam, Khaled Al-Kharouf, Mo’ath Bani Ali, Moath Ahmad Albliwi, Rawan Al-Hamarsheh, Mohammad Iswaid, Ahmad Albanna, Ahmad Enjadat, Mohammad Abu Orabi Al-Adwan, Khaled Dibbeh, Ez-Aldeen Abu Shareah, Anas Hamdan, Aiman Suleiman

**Affiliations:** 1 Department of Anesthesia and Intensive Care Unit, The Hashemite University, Zarqa 13115, Jordan; sager@hu.edu.jo; 2 School of Medicine, The University of Jordan, Amman 11942, Jordan; fuad.41994@gmail.com (F.M.Z.); moathmegdadi@gmail.com (M.B.A.); moathelbalawe2018@yahoo.com (M.A.A.); rawanmdhamarsheh@gmail.com (R.A.-H.); mohd_iswaid@hotmail.com (M.I.); dr.albanna10@gmail.com (A.A.); dr.mohaladwan@hotmail.com (M.A.O.A.-A.); 3 Department of Anesthesia and Intensive Care, Alabdali Clemenceau Hospital, Amman 11190, Jordan; alaliobada.ane@gmail.com; 4 School of Medicine, The Hashemite University, Zarqa 13115, Jordan; alazzamahmed@yahoo.com; 5 Southampton Orthopedics: Centre for Arthroplasty and Revision Surgery, University Hospital Southampton, Tremona Road, Southampton SO16 6YD, UK; kfk990@gmail.com; 6 Department of Internship Program, Jordan University Hospital, Amman 11942, Jordan; ahmadamjse1996@gmail.com; 7 Leicester University Hospitals, P.O. Box 7853, Leicester LE1 9WW, UK; khaled.dibbeh@uhl-tr.nhs.uk; 8 Accident and Emergency Department, The Princess Alexandra Hospital NHS Trust, Hamstel Road, Harlow CM20 1QX, UK; dr.ezzaldeen2017@gmail.com; 9 Department of Anesthesia and Intensive Care Unit, Istishari Hospital, Amman 11184, Jordan; ans.ham88@hotmail.com; 10 Department of Anesthesia, Intensive Care and Pain Management, Harvard Medical School, Beth Israel Deaconess Medical Center, Boston, MA 02215, USA; asuleima@bidmc.harvard.edu

**Keywords:** neuropathic pain, quantum tunneling, ion channels, quantum biology, quantum medicine, quantum conductance, depolarization, ephaptic coupling

## Abstract

Neuropathic pain is a challenging complaint for patients and clinicians since there are no effective agents available to get satisfactory outcomes even though the pharmacological agents target reasonable pathophysiological mechanisms. This may indicate that other aspects in these mechanisms should be unveiled to comprehend the pathogenesis of neuropathic pain and thus find more effective treatments. Therefore, in the present study, several mechanisms are chosen to be reconsidered in the pathophysiology of neuropathic pain from a quantum mechanical perspective. The mathematical model of the ions quantum tunneling model is used to provide quantum aspects in the pathophysiology of neuropathic pain. Three major pathophysiological mechanisms are revisited in the context of the quantum tunneling model. These include: (1) the depolarized membrane potential of neurons; (2) the cross-talk or the ephaptic coupling between the neurons; and (3) the spontaneous neuronal activity and the emergence of ectopic action potentials. We will show mathematically that the quantum tunneling model can predict the occurrence of neuronal membrane depolarization attributed to the quantum tunneling current of sodium ions. Moreover, the probability of inducing an ectopic action potential in the axons of neurons will be calculated and will be shown to be significant and influential. These ectopic action potentials are generated due to the formation of quantum synapses which are assumed to be the mechanism behind the ephaptic transmission. Furthermore, the spontaneous neuronal activity and the emergence of ectopic action potentials independently from any adjacent stimulated neurons are predicted to occur according to the quantum tunneling model. All these quantum mechanical aspects contribute to the overall hyperexcitability of the neurons and to the pathogenesis of neuropathic pain. Additionally, providing a new perspective in the pathophysiology of neuropathic pain may improve our understanding of how the neuropathic pain is generated and maintained and may offer new effective agents that can improve the overall clinical outcomes of the patients.

## 1. Introduction

Neuropathic pain is a challenging entity in clinical practice for both patients and clinicians [1]. Many pathologies are involved in the pathogenesis of neuropathic pain. These pathologies can be classified into central and peripheral categories. The central category involves the brain and the spinal cord such as cerebrovascular disease, neurodegenerative diseases such multiple sclerosis and Parkinson disease, spinal cord injury, syringomyelia, and transverse myelitis [1]. The peripheral category involves diabetes, infection (Herpes Zoster), nerve compression, nerve trauma, autoimmune diseases, cancer and its related chemotherapy and inherited channelopathies [1]. Furthermore, there are many complex pathophysiological mechanisms that generate neuropathic pain, which leave this disease with difficult management and control [1,2,3,4,5,6,7,8,9,10,11,12,13]. These mechanisms include neuronal membrane depolarization, the spontaneous firing of the neurons, and the neuronal ephaptic coupling [1,2,3,4,5,6,7,8,9,10,11,12,13]. The membrane depolarization that has been observed in the injured neurons can explain the hyperexcitability because in this case the neurons can reach the threshold of action potential more easily [2,3,4,5]. Moreover, the spontaneous firing of injured neurons and the emergence of ectopic action potentials are key features in the pathophysiology of neuropathic pain [1,6,7,8,9]. Another important aspect that contributes to the maintenance of neuropathic pain is the ephaptic interactions between injured fibers because this type of transmission implies the communication of neurons without the requirement of the usual chemical and electrical synapses [10,11,12,13].

Quantum biology is a new emerging and promising field that exploits the principles of quantum mechanics such as quantum tunneling, quantum entanglement, and many others to understand and delineate poorly understood actions, processes, and events in the biological systems [14]. Recently, the authors of this review [15] focused on the potential capability of quantum biology to cause a significant improvement in the clinical outcomes of several challenging diseases.

In the present study, we aim to revisit the previous three major mechanisms of the pathophysiology of neuropathic pain from a quantum mechanical perspective. This aim is accomplished by utilizing the model of ions quantum tunneling through the closed gates of voltage-gated channels. This quantum model has been proposed before and used in different contexts to explain several physiological and pathophysiological actions and processes [16,17,18,19,20]. This quantum model is based on one of the major consequences of quantum mechanics, which is quantum tunneling. The quantum tunneling describes the ability of quantum particles such as electrons, protons, atoms, and ions to pass through a barrier whose energy is higher than the energy of the particle. Classically, this particle cannot pass through this barrier because it does not have enough energy to overcome it. This wired quantum behavior is attributed to the wave nature of the particle when it is described according to the principles of quantum mechanics. Hence, this phenomenon can be applied on the voltage-gated channels since they possess a closed gate which can be illustrated as a potential barrier that blocks the permeations of ions. In this case, ions can be described as quantum particles and the closed gate as the barrier that has higher energy than the ions. Accordingly, the quantum tunneling model predicts that closed channels have a quantum permeability or quantum conductance mediated via quantum tunneling. This quantum model showed the potential ability to provide reasonable and comprehensive explanations for several physiological and pathological conditions. For example, the model could explain how the myelin sheath can increase the spatiotemporal fidelity of action potential signals by eliminating the quantum tunneling of potassium ions [19]. Furthermore, it could provide a comprehensive understanding of the pathophysiology of referred pain and phantom limb pain based on the mathematical modeling of quantum tunneling of ions [18,20]. Our motivation behind using the quantum tunneling model for neuropathic pain is to improve the unsatisfactory clinical outcomes of using the currently available medications [21], hence providing a new perspective may provide the opportunity to produce more effective medications to get more satisfactory clinical outcomes. Another motivation is the ability of the quantum tunneling model to provide a more comprehensive and reasonable explanation for the electrophysiological features observed in the neurons of the neuropathic pain, especially as some of these features cannot be fully understood by the classical methods or do not have a clear mechanistic basis [1,2,3,4,5,6,7,8,9,10,11,12,13,21,22]. The quantum mechanical aspects presented in this study will provide new mechanisms not discussed before to explain the hyperexcitability of the injured neurons such as the quantum tunneling-induced membrane depolarization, give consistent and reasonable explanations for elusive pathophysiological events such as ephaptic interactions between neurons by proposing the idea of quantum synapses, and show that the quantum tunneling of ions can play a more significant role than the pure thermal mechanism to generate spontaneous ectopic action potentials by applying the idea of thermally-assisted quantum tunneling.

## 2. The Mathematical Model

### 2.1. The Quantum Tunneling of Ions and the Quantum Conductance

In the present study, the mathematical model of ions quantum tunneling will be applied to investigate the quantum aspects of the pathogenesis of neuropathic pain. It will be applied on the voltage-gated sodium channels, which are strongly involved in the pathophysiology of neuropathic pain [8,9] to explain how membrane depolarization is induced from a quantum mechanical point of view and to show that neurons can trigger a spontaneous ectopic action potential. Additionally, the quantum tunneling model will be applied on the voltage-gated potassium channels to propose the idea of quantum synapses between neurons. The voltage-gated channels possess an intracellular hydrophobic closed gate that seals off the permeation of ions [23,24,25,26]. Accordingly, this closed gate functions as an energy barrier that blocks the passage of ions. In this case, the quantum tunneling phenomenon is applicable and it is useful to investigate the properties of ion passage when the gate is closed.

The symmetric Eckart potential will be used in this study to scrutinize the quantum tunneling probability of ions through the closed gate of voltage-gated channels. The symmetric Eckart potential can be mathematically represented by the following function [27,28,29]:

(1) $$U(x)=\frac{G}{\cosh^{2}(\frac{x}{L})}$$

where *U(x)* is the energy barrier of the closed gate, *G* is the barrier height of the closed gate, *x* is the position of the ion in the gate, and *L* is the ‘gate length’ at which U(L)=0.42G.

**Figure 1:**
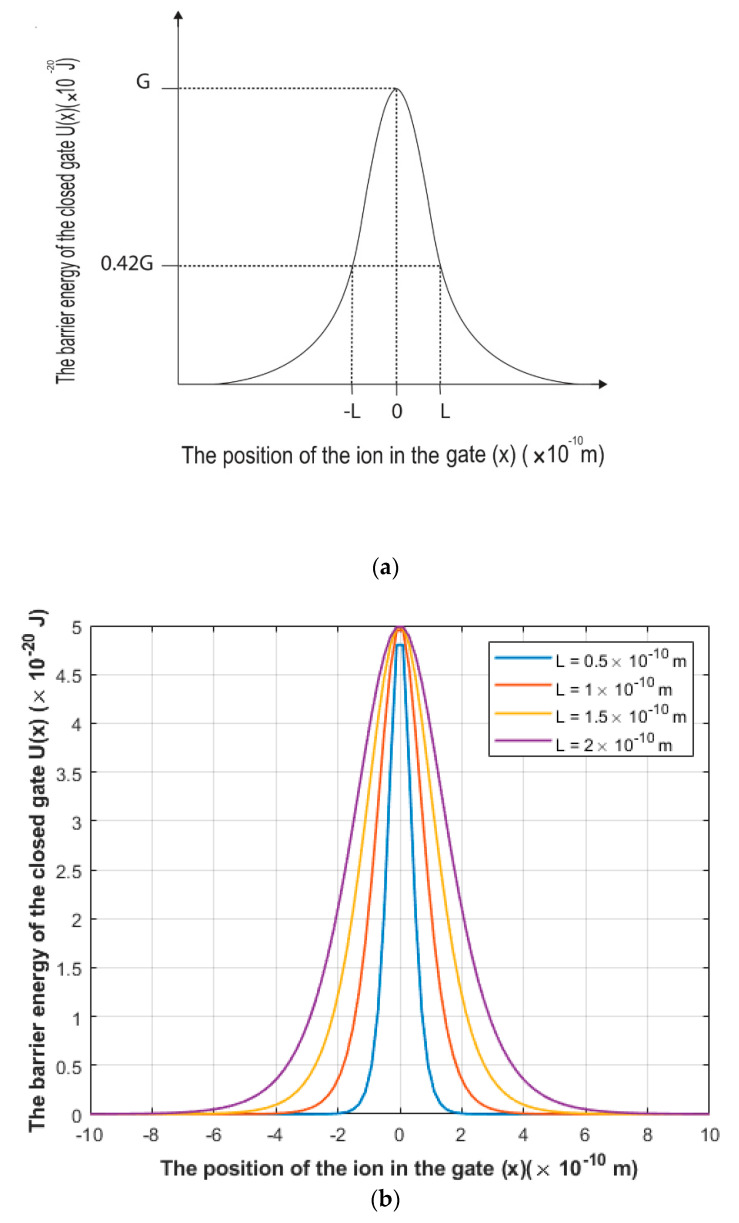
(**a**) A schematic representation of the symmetric Eckart potential. (**b**) Real plotting of the symmetric Eckart potential at different values of gate length *L*.

The graphical representation of the symmetric Eckart potential can be found in Figure 1.

We chose the symmetric Eckart potential to estimate the tunneling probability of ions through the closed gates because the experimental observations of the energy barrier of the hydrophobic gate based on the potential mean forces (PMF) of ions [30,31,32,33,34] reveals a similar pattern as in Figure 1. Moreover, there is no concrete mathematical equation that can describe how the energy barrier changes with respect to the ion’s position because what we have from the literature with respect to the energy barrier of the closed gate is an experimental measurement without a clear mathematical framework. Therefore, the symmetric Eckart potential can mimic and approximate the barrier shapes obtained experimentally.

Accordingly, the quantum tunneling probability through the symmetric Eckart potential can be calculated using the following equation [27,28,35]:

(2) $$T_{Q}=e^{\alpha L(\sqrt{G}-\sqrt{KE})}$$

where $\alpha =\frac{-\sqrt{8\pi^{2}m}}{\hbar }$; where *m* is the mass of the ion ($m_{Na}=3.8\times 10^{-26}$ Kg and $m_{K}=6.5\times 10^{-26}$ Kg), ℏ is the reduced Planck constant ($1.05\times 10^{-34}$ Js), *L* is the length of the gate at which U(L)=0.42G, *G* is the barrier height of the closed gate, and *KE* is the kinetic energy of the ion.

**Figure 2:**
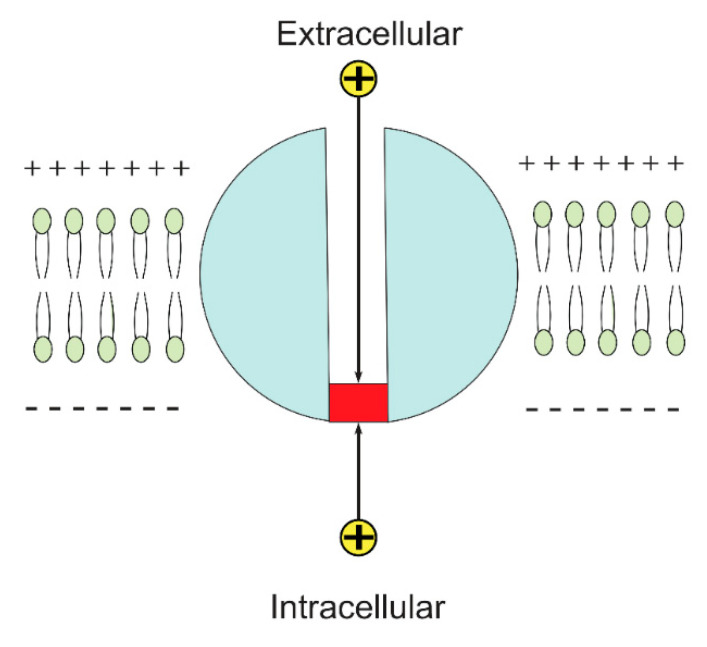
The figure represents a schematic diagram of the closed voltage-gate channel in which the closed gate, shown by the red color, is located at the intracellular end. The extracellular ion goes through the membrane potential until hitting the closed gate, while the intracellular ion hits the closed gate before going through the membrane potential.

There are extracellular and intracellular ions and they are different in terms of kinetic energy KE [16,17] due the influence of the membrane potential. As the usual location of the closed activation gate is at the intracellular end [23,24,25], then the extracellular ions will go through the membrane potential, which is negative inside with regard to outside, acquiring kinetic energy equal to $qV_{m}$ until reaching the intracellular closed gate, in addition to the average thermal energy $\frac{1}{2}K_{B}T$, while intracellular ions will hit the intracellular closed gate having only the average thermal energy $\frac{1}{2}K_{B}T$. See Figure 2.

**Figure 3:**
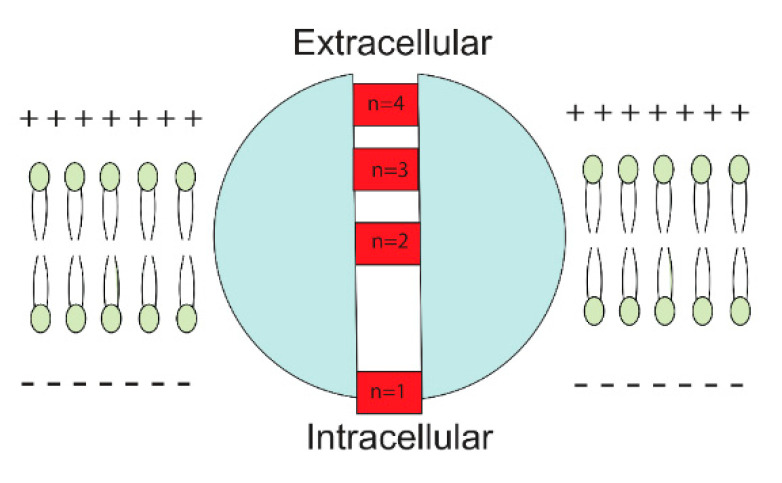
The figure represents the possible locations of the closed gate by assigning *n* values from 1 to 4.

However, the quantum tunneling model can be applied on the closed gates, which include the closed activation and closed inactivation gates. Moreover, the closed inactivation gate can be located at sites other than the intracellular end [36,37,38]. Therefore, to account for the different locations of the closed gate and its influence on the kinetic energy of the extracellular ions, we will assign *n* values from 1 to 4. See Figure 3.

Accordingly, the kinetic energies of the extracellular and intracellular ions, respectively, are:

(3) $$KE_{e}=\frac{qV_{m}}{n}+\frac{1}{2}K_{B}T$$

(4) $$KE_{i}=\frac{1}{2}K_{B}T$$

where *q* is the charge of the ion, V_m_ is the membrane potential, *n* is the location of the closed gate, $K_{B}$ is the Boltzmann’s constant ($1.38\times 10^{-23}$ J/K), and *T* is the body temperature (310 K).

Thus, the quantum tunneling probabilities of the extracellular and intracellular ions are, respectively:

(5) $$T_{Q(e)}=e^{\alpha L(\sqrt{G}-\sqrt{\frac{qV_{m}}{n}+\frac{1}{2}K_{B}T})}$$

(6) $$T_{Q(i)}=e^{\alpha L(\sqrt{G}-\sqrt{\frac{1}{2}K_{B}T})}$$

Since there is a probabilistic passage through the closed gate via quantum tunneling, then it is expected to observe a quantum conductance. Hence, the quantum unitary conductance of the closed voltage-gated channels can be calculated using the following equation [39,40,41]:

(7) $$C_{Q}=\frac{q^{2}}{h}T_{Q}$$

where $C_{q}$ is the quantum unitary conductance, *q* is the ion’s charge ($1.6\times 10^{-19}$ C), *h* is the Planck constant ($6.6\times 10^{-34}$ Js), and $T_{Q}$ is the tunneling probability. The unit of quantum unitary conductance will be Siemens (S).

Additionally, when there are certain numbers of ion channels in certain surface areas of the biological membrane, the quantum membrane conductance can be calculated using the following equation [42,43]:

(8) $$MC_{Q}=C_{Q}D$$

where $MC_{Q}$ is the quantum membrane conductance and *D* is the channel density (channels/cm^2^). The unit of quantum membrane conductance will be mS/cm^2^.

### 2.2. The Quantum Tunneling-Induced Membrane Depolarization and The Quantum Tunneling Current

Eventually, to assess the influence of the quantum conductance on the resting membrane potential, the Goldman–Hodgkin–Katz (GHK) equation is used.

The classical version of the GHK equation, which does not involve the quantum conductance, is [42,43]:

(9) $${\left[Na\right]}_{e}MC_{Na}+{\left[K\right]}_{e}MC_{K}=e^{\frac{-FV_{m}}{RT}}({\left[Na\right]}_{i}MC_{Na}+{\left[K\right]}_{i}MC_{K})$$

where ${\left[Na\right]}_{e}=142$ mmol/L [42,43] is the extracellular sodium concentration, ${\left[K\right]}_{e}=4$ mmol/L [42,43] is the extracellular potassium concentration, ${\left[Na\right]}_{i}=14$ mmol/L [42,43] is the intracellular sodium concentration, ${\left[K\right]}_{i}=140$ mmol/L [42,43] is the intracellular potassium concentration, $MC_{Na}=0.022$ mS/cm^2^ [42,43] is the leaky membrane conductance of sodium ions,$MC_{K}=0.5$ mS/cm^2^ [42,43] is the leaky membrane conductance of potassium ions, *F* is Faraday’s constant (96,485.33 C/mol), *R* is the gas constant (8.31 J/Kmol), *T* is the body temperature (310 K), and $V_{m}$ is the membrane potential. If the previous values are substituted in Equation (9), the membrane potential will be $V_{m}=0.07$ V.

On the other hand, to investigate the influence of quantum tunneling of ions and their quantum conductance on membrane potential, the quantum version of the GHK equation will be used [44]:

(10) $${\left[Na\right]}_{e}MC_{Na}+{\left[K\right]}_{e}MC_{K}+{\left[ion\right]}_{e}MC_{Q(ion)e}=e^{\frac{-FV_{m}}{RT}}({\left[Na\right]}_{i}MC_{Na}+{\left[K\right]}_{i}MC_{K}+{\left[ion\right]}_{i}MC_{Q(ion)i})$$

where ${\left[ion\right]}_{e}$ is the extracellular concentration of the ion on which the quantum tunneling model is applied, ${\left[ion\right]}_{i}$ is its intracellular concentration, $MC_{Q(ion)e}$ is the quantum membrane conductance of the extracellular ion, and $MC_{Q(ion)i}$ is the quantum membrane conductance of the intracellular ion.

**Figure 4:**
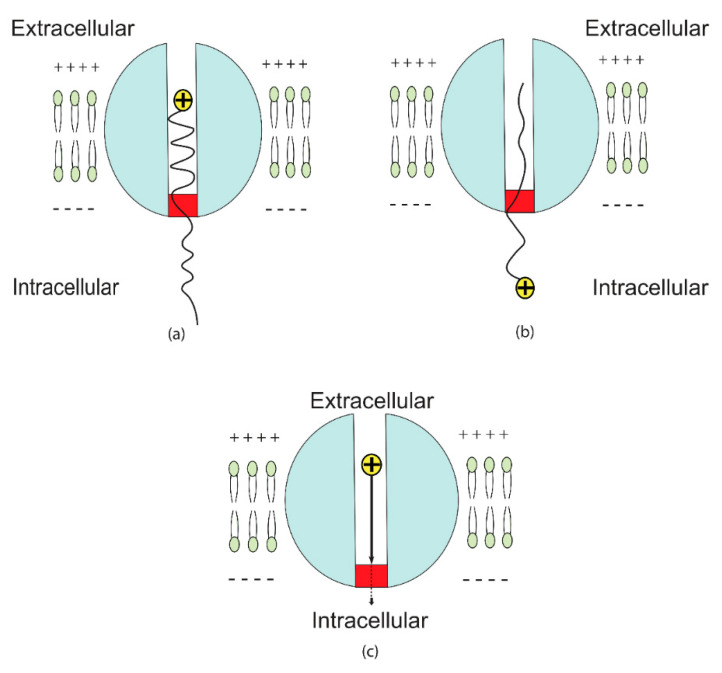
(**a**) The quantum tunneling of the extracellular ion through the closed gate. (**b**) The quantum tunneling of the intracellular ion. The quantum tunneling of the extracellular sodium ion has higher probability if it is compared with the quantum tunneling of the intracellular sodium ion. This is represented by the higher wave amplitude of the extracellular ion after tunneling through the closed gate, while the intracellular sodium ion has lower wave amplitude. The discrepancy between the extracellular and intracellular ions in tunneling probability is due to the difference in the kinetic energy. The extracellular ions have higher kinetic energy, which is represented by the shorter wavelength and the intracellular ions have lower kinetic energy, which is represented by the longer wavelength. (**c**) As a result, there will be a net inward quantum tunneling current of ions that has the tendency to depolarize the membrane potential.

Furthermore, an important aspect will be covered in this paper, which is the unitary quantum tunneling current. It can be calculated using the following equation:

(11) $$I_{T-channel}=I_{T-e}-I_{T-i}$$

where $I_{T-channel}$ is the unitary quantum tunneling current of channel, $I_{T-e}$ is the extracellular tunneling current, and $I_{T-i}$ is the intracellular tunneling current. The intracellular tunneling current can be neglected since the tunneling probability of intracellular ions is small if it is compared with that of extracellular ions and if Equations (5) and (6) are substituted with the same values. Hence, it can be said that the major source of the quantum unitary current is the quantum tunneling of the extracellular ions. See Figure 4.

Therefore, the quantum unitary tunneling current of the closed channel can be expressed by the following equation:

(12) $$I_{T-channel}=\frac{q^{2}V_{m}}{h}e^{\alpha L(\sqrt{G}-\sqrt{\frac{qV_{m}}{n}+\frac{1}{2}K_{B}T})}$$

The unit of quantum tunneling current is Ampere (A).

### 2.3. The Formation of Quantum Synapses between The Axons as a Mechanism for Ephaptic Coupling

The second aspect that will be investigated in the context of the quantum tunneling model is the ephaptic transmission. This type of transmission has been observed, which implies interactions between the axons of the neurons without chemical or electrical synapses [10,11,12,45,46,47]. Thus, if one neuron fires, then an adjacent unstimulated neuron will be triggered to transmit an action potential. However, this type of transmission does not have a clear mechanism because the voltage changes that are induced by neuronal firing are too small to trigger an action potential [45,47]. However, when this type of transmission is investigated by the quantum tunneling model, it can explain the ephaptic coupling without the requirement of large changes in the membrane potential or even the requirement of large changes in the extracellular potassium concentration. This quantum aspect has been utilized before to explain the pathophysiology of phantom limb pain and tinnitus [20,35]. The idea is based on the ability of potassium ions, which exit to the extracellular fluid during action potentials, to tunnel through the closed channels in the membrane of the neighboring unstimulated neurons.

**Figure 5:**
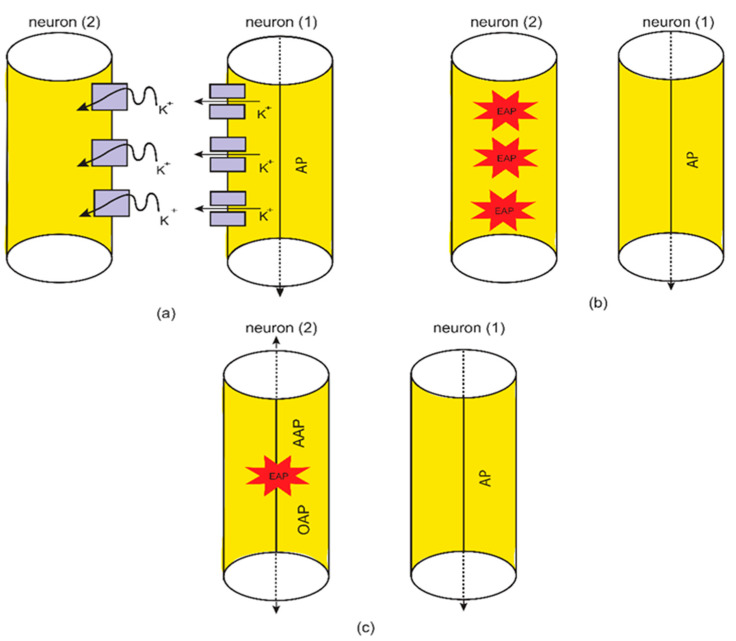
A schematic diagram of the ephaptic transmission from the quantum mechanical point of view or the quantum synapse. (**a**): the quantum tunneling of potassium ions through the closed channels in the membrane of adjacent unstimulated neuron. (**b**): the generation of ectopic action potentials (EAPs). (**c**): antidromic action potential (AAP) and orthodromic action potential (OAP) are generated.

The basic idea behind the formation of a “quantum synapse” between axons is that when an action potential is transmitted through a neuron, there will be a probability that this stimulated neuron will induce an ectopic action potential (EAP) in an adjacent unstimulated neuron. This ectopic action potential induction is achieved via the quantum tunneling of potassium ions through the closed potassium channels, which are exposed upon demyelination after being covered by the myelin sheath [48,49,50,51]. This implies that the formation of quantum synapses is enhanced among unmyelinated or demyelinated neurons because more potassium channels are available for quantum tunneling of potassium ions. If an ectopic action potential is induced, then it is expected to detect both orthodromic action potential (OAP) propagation and antidromic action potential (AAP) propagation [11,52,53]. See Figure 5.

While the action potential is being generated, there will be an increase in the extracellular potassium concentration. We assume that there are $1.37\times 10^{6}$ potassium ions per 314 µm^2^ of the neuronal membrane ($4.36\times 10^{3}$ ions/µm^2^) [18,43], which exit during an action potential. Furthermore, a neuron with a length L = 100 µm and axonal radius r = 0.5 µm can yield a surface area of 314 µm^2^ and an intracellular neuronal volume of 78.5 µm^3^ (assuming that the neuron has the shape of a cylinder). Therefore, the extracellular volume that potassium ions diffuse into can be estimated to be 52.6 µm^3^ (assuming the ratio between the extracellular and intracellular volumes is 0.67 [43,54]). Accordingly, the increase in the extracellular potassium concentration can be calculated using the following equation:

(13) $${\left[K\right]}_{AP}=\frac{N_{AP}}{N_{A}V_{E}}$$

where ${\left[K\right]}_{AP}$ is the magnitude of the increase in the extracellular potassium concentration during the action potential, $N_{AP}$ is the number of potassium ions that exit to the extracellular compartment per unit surface area and per action potential, $N_{A}$ is Avogadro’s number, and $V_{E}$ is the volume of the extracellular compartment where potassium ions exit to.

Based on our previous example, we can substitute the parameters $N_{AP}=1.37\times 10^{6}$ potassium ions (corresponding to 314 µm^2^), $V_{E}=52.6$ µm^3^, and $N_{A}=6.02\times 10^{23}$ mol^−1^ into Equation (13) to get ${\left[K\right]}_{AP}=4.3\times 10^{-2}$ mmol/L. We provide this example to make it easier to follow the subsequent numerical results and to facilitate understanding of the concept of a quantum synapse between axons.

When potassium ions exit to the extracellular compartment, the average number of potassium ions $N_{K}$ that can hit a single closed channel in the membrane of an adjacent unstimulated neuron can be calculated using the following equation:

(14) $$N_{K}=\frac{N_{AP}}{D}$$

where *D* is the channel density and $N_{AP}$ is the number of potassium ions that exit through a specific surface area of the neuronal membrane. When Equation (14) is applied, it is important to make sure that the surface area unit in the quantities of $N_{AP}$ and *D* is the same. According to our previous example, when $N_{AP}=4.36\times 10^{3}$ ions/µm^2^ (which corresponds to $N_{AP}=1.37\times 10^{6}$ ions/314 µm^2^) and $D=10^{2}$ channels/µm^2^ [42,43] (which corresponds to $D=10^{10}$ channels/cm^2^), $N_{K}=44$ ions, which is the number of potassium ions that hit a single closed channel. Thus, the number of potassium ions $N_{K}$ corresponds to the change in the extracellular potassium concentration of $4.3\times 10^{-2}$ mmol/L. As the change in the extracellular potassium concentration increases, the average number of potassium ions hitting the channel increases.

If this minute concentration is substituted in Equation (9), there will be almost no change in the membrane potential of the neurons. However, we will reveal that the idea of the quantum synapse permits for this minor change in potassium concentration to depolarize the membrane and induce an ectopic action potential. This is a unique feature of the quantum synapse that makes it distinct from the classical electrical communication between neurons. Next, we will calculate the threshold value of quantum tunneling $T_{Q(Thr)}$ that gives a threshold value of quantum conductance that can depolarize the membrane to the threshold value of potential $V_{m(Thr)}$, inducing an ectopic action potential. The $V_{m(Thr)}$ will be assumed to be 55 mV.

The following equation can be used to obtain a relationship between $T_{Q(Thr)}$ and ${\left[K\right]}_{AP}$:

(15) $$MC_{Na}{\left[Na\right]}_{e}+MC_{K}{\left[K\right]}_{e}+{\left[K\right]}_{AP}MC_{Q-K(e)}=e^{\frac{-FV_{m(Thr)}}{RT}}(MC_{Na}{\left[Na\right]}_{i}+MC_{K}{\left[K\right]}_{i})$$

For the sake of simplicity, we will assume that one channel of the total channels in 1 µm^2^ is enough to depolarize the membrane potential to the threshold value. Therefore, $D=10^{8}$ channels/cm^2^ (which corresponds to 1 channel/µm^2^) will be substituted in Equation (15). Accordingly, by substituting the values of concentrations and conductance in Equation (15), the relationship between $T_{Q(Thr)}$ and ${\left[K\right]}_{AP}$ can be obtained:

(16) $$T_{Q(Thr)}=\frac{9.82\times 10^{-7}}{{\left[K\right]}_{AP}}$$

Substituting Equation (13) in Equation (16):

(17) $$T_{Q(Thr)}=\frac{9.82\times 10^{-7}N_{A}V_{E}}{N_{AP}}$$

Substituting $N_{A}=6.02\times 10^{23}$ mol^−1^ and $V_{E}=52.6$ µm^3^, then:

(18) $$T_{Q(Thr)}=\frac{31.1}{N_{AP}}$$

where $N_{AP}$ is the number of potassium ions that exit from a surface area of 314 µm^2^, which corresponds to $V_{E}=52.6$ µm^3^, per action potential.

Substituting Equation (14) in Equation (18):

(19) $$T_{Q(Thr)}=\frac{31.1}{314N_{K}D}=\frac{9.9\times 10^{-2}}{N_{K}D}$$

where *D* is the channels density (channels/µm^2^).

Equations (16)–(19) will help us to investigate how the threshold value of quantum tunneling of potassium ions changes with respect to the potassium concentration, number of potassium ions that exit from a specific surface area of neurons, and the number of potassium ions hitting a single closed channel, respectively.

If ${\left[K\right]}_{AP}=4.3\times 10^{-2}$ mmol/L is substituted into Equation (16), then the threshold value of quantum tunneling $T_{Q(Thr)}=2.24\times 10^{-5}$. This means that if at least one channel in a surface area of 1 µm^2^ is required to induce an action potential, then at least a fraction of $2.24\times 10^{-5}$ from the total potassium ions hitting the channel must tunnel through the closed gate to depolarize the membrane potential sufficiently to induce an ectopic action potential. As was explained before, this change in membrane potential corresponds to around 44 potassium ions, which hit a single closed channel. Then, if at least one potassium ion from the total 44 potassium ions tunnels through the closed gate, then the minimum tunneling fraction will be $\frac{1}{44}=2.27\times 10^{-2}$. If this minimum fraction is compared with $T_{Q(Thr)}=2.24\times 10^{-5}$, which represents the minimum tunneling fraction required to induce an action potential from at least one channel in 1 µm^2^, it is clear that the process of tunneling can induce an ectopic action potential since $2.27\times 10^{-2}>2.24\times 10^{-5}$. Therefore, we aim to calculate the probability of reaching this significant fraction of tunneling based on the actual tunneling probability, as shown in Equation (5). Since the action potential is propagated through the neuron, there will be many chances available for potassium ions to tunnel through the closed channels in the membrane of unstimulated unmyelinated/demyelinated neurons. This implies that along the surface area available for potassium ion tunneling, there will be a probability that at least one potassium ion from the total number hitting a channel (e.g., 44 ions here) will succeed to tunnel and trigger an ectopic action potential.

To calculate the probability of the induction of an ectopic action potential in an adjacent unstimulated neuron by another neuron carrying the signal of the action potential (AP), the Bernoulli trials equation can be used as follows:

(20) $$P(Z)=\frac{N!P^{Z}{\left(1-P\right)}^{N-Z}}{(N-Z)!Z!}$$

where *Z* is the number of trials that must be achieved successfully, *N* is the total number of available trials, *P* is the probability of achieving a successful trial, and *P(Z)* is the probability of obtaining *Z* number of successful trials. When Z = 0, then:

(21) $$P(0)={\left(1-P\right)}^{N}$$

Therefore, if an action potential is transmitted through a neuron and an increase in the extracellular potassium ions happens, then the probability that at least one potassium ion from the total number $N_{K}$ hitting a single closed channel will succeed and tunnel through the closed gate can be calculated using the following equation:

(22) $$P_{1}=1-{\left(1-T_{Q(K(e))}\right)}^{N_{K}}$$

where $P_{1}$ is the probability of triggering an ectopic action potential by one channel via quantum tunneling of at least one potassium ion and $T_{Q(K(e))}$ is the quantum tunneling probability of extracellular potassium ions delineated in Equation (5).

Furthermore, the probability that at least one closed channel from the total number of channels $D_{\mu m^{2}}$ in 1 µm^2^ is tunneled by at least one potassium ion can be calculated using the following equation:

(23) $$P_{2}=1-{\left(1-P_{1}\right)}^{D_{\mu m^{2}}}$$

where $P_{2}$ is the probability of triggering an action potential in a surface area of 1 µm^2^ via quantum tunneling of at least one potassium ion through at least one closed channel.

Eventually, the probability of triggering an ectopic action potential in at least one area of 1 µm^2^ from the total number of surface areas $N_{\mu m^{2}}$ can be calculated by the following equation:

(24) $$P_{3}=1-{\left(1-P_{2}\right)}^{N_{\mu m^{2}}}$$

where $P_{3}$ is the probability of triggering an ectopic action potential in 1 µm^2^ from the total number of surface areas $N_{\mu m^{2}}$. $P_{3}$ represents the eventual probability of ectopic action potential induction along the surface area available for the quantum tunneling of potassium ions. The total number of surface areas of 1 µm^2^ can be calculated using the following equation:

(25) $$N_{\mu m^{2}}=\frac{A}{1\mu m^{2}}$$

where *A* is the surface area (in µm^2^) that has been demyelinated or it is actually unmyelinated and is available for quantum tunneling through its exposed potassium channels. For example, if $A=10^{-10}$ m^2^ = 100 µm^2^, then $N_{\mu m^{2}}=100$. This means that there are 100 areas available for the quantum tunneling of potassium ions to trigger an ectopic action potential.

$P_{3}$ represents the probability of inducing an action potential in an unstimulated neuron when it is exposed to a neuron carrying a single action potential. Hence, when a neuron is exposed to λ number of neurons carrying κ number of action potentials, then the exposed unstimulated neuron can become stimulated with ω number of induced action potentials:

(26) $$\omega =P_{3}\lambda \kappa$$

### 2.4. The Quantum Tunneling-Induced Spontaneous Neuronal Firing

**Figure 6:**
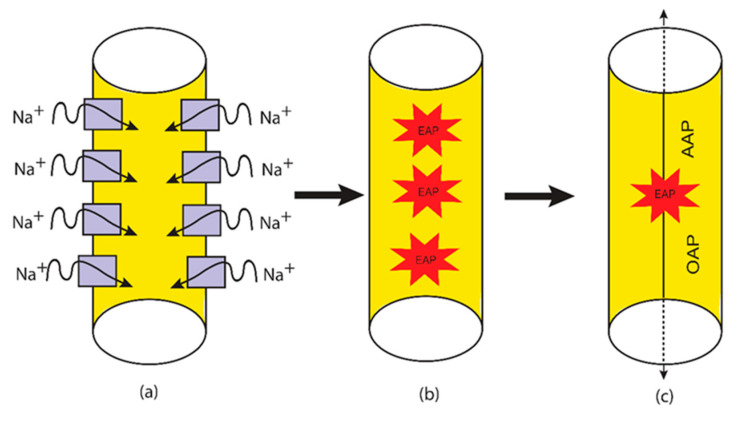
A schematic diagram of the quantum tunneling-induced spontaneous firing and the generation of ectopic action potentials, which are mediated by the quantum tunneling of sodium ions. (**a**): the quantum tunneling of sodium ions through the closed channels. (**b**): the generation of ectopic action potentials (EAPs). (**c**): antidromic action potential (AAP) and orthodromic action potential (OAP) are generated.

The third aspect is the spontaneous firing that is mediated by the quantum tunneling of sodium ions. This aspect will be discussed and linked to the neuropathic pain. This idea implies that under thermal fluctuations, the closed channels can get different amounts of thermal energy in a probabilistic way. The unique feature here is that this thermal energy is not intended to open the closed gate rather it is intended to reduce the barrier energy of the closed gate transiently so that the quantum tunneling of sodium ions is augmented. This thermally assisted quantum tunneling can result in a significant tunneling current of sodium ions that can depolarize the membrane potential to threshold of action potential induction. See Figure 6.

Accordingly, the expectant value or the average value of quantum tunneling probability due to the thermal fluctuations can be mathematically represented by the following equation:

(27) $$\langle T_{Q}\rangle =\frac{1}{\beta }\int_{0}^{E\leq G-KE}e^{\frac{-E}{\beta }+\alpha L(\sqrt{G-E}-\sqrt{KE})}dE$$

where $\beta =K_{B}T$, $\alpha =\frac{-\sqrt{8\pi^{2}m}}{\hbar }$ and *E* is the thermal assisting energy that can lower the barrier energy of the closed gate.

As a result, the expectant or the average value of quantum membrane conductance due to the thermal fluctuations:

(28) $$\langle MC_{Q}\rangle =\delta \int_{0}^{E\leq G-KE}e^{\frac{-E}{\beta }+\alpha L(\sqrt{G-E}-\sqrt{KE})}dE$$

where $\delta =\frac{Dq^{2}}{h\beta }$. The unit of $\langle MC_{Q}\rangle$ will be mS/cm^2^.

To assess the influence of the thermal fluctuations on the quantum tunneling-induced spontaneous firing, the following equation can be used:

(29) $${\left[Na\right]}_{e}MC_{Na}+{\left[K\right]}_{e}MC_{K}+{\left[ion\right]}_{e}\langle MC_{Q(ion)e}\rangle =e^{\frac{-FV_{m}}{RT}}({\left[Na\right]}_{i}MC_{Na}+{\left[K\right]}_{i}MC_{K}+{\left[ion\right]}_{i}\langle MC_{Q(ion)i}\rangle )$$

Furthermore, we aim to show the differences between the spontaneous firing induced by the pure thermal fluctuations and the quantum tunneling-induced spontaneous firing. Hence, the following equation can be used to assess the influence of the thermal fluctuations on the classical opening of the closed channels and thus on the membrane potential:

(30) $${\left[Na\right]}_{e}MC_{Na}+{\left[K\right]}_{e}MC_{K}+{\left[ion\right]}_{e}\frac{DC_{open(mS)}}{1+e^{\frac{G}{\beta }}}=e^{\frac{-FV_{m}}{RT}}({\left[Na\right]}_{i}MC_{Na}+{\left[K\right]}_{i}MC_{K}+{\left[ion\right]}_{i}\frac{DC_{open(mS)}}{1+e^{\frac{G}{\beta }}})$$

where $C_{open(mS)}$ is the conductance of single channel by the unit of mS when it is open, which is within an order of magnitude $10^{-12}$ S [42,43].

Equations (29) and (30) will be applied on voltage-gated sodium channels and their corresponding sodium ions.

**Figure 7:**
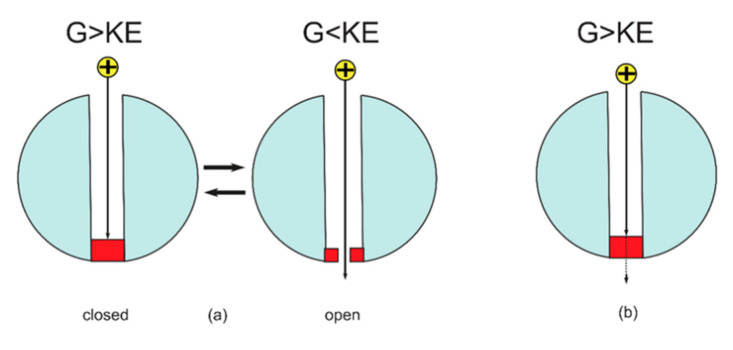
The figure represents a schematic diagram of ion passage through the ion channel according to the classical and quantum tunneling models. (**a**) The classical model: when the barrier height of the gate is higher than the kinetic energy of the ions, the gate is considered to be closed and the permeation is blocked, while when the barrier height of the gate is lower than the kinetic energy of the ion, the gate is considered to be open and the permeation is allowed. (**b**) The quantum tunneling model: the ion can permeate via quantum tunneling through the gate even though its energy barrier is higher than the kinetic energy of the ion.

At the level of single voltage-gated channel, the classical spontaneous firing depends on the transient opening of the closed channel and the switch between the open and the closed states, while the quantum spontaneous firing depends on the persistent tunneling current that is generated through the closed channel. See Figure 7.

## 3. Results

In the present study, we focused on two major pathological molecular events that occur during the pathogenesis of neuropathic pain. The first event is that the barrier height of the closed gate *G* drops due the pathological causes mentioned before because it has been found that inflammation, trauma, and ischemia or any cause can affect the integrity of the cellular membrane and can render these channels leaky [55,56,57]. This leakiness is due to the drop in the energy barrier height of the closed gates indicated by the shift in the activation and inactivation curves [55,56,57]. Moreover, it is argued that voltage-gated sodium channels have a lower activation threshold [8,9], indicating that less energy is required to activate them if they are compared with the normal ones [8,9]. Additionally, the inflammatory mediators released during the pathogenesis of neuropathic pain such as IL-1 beta sensitize and potentiate the voltage-gated channels, which explains their pathological role [8,9,22,58,59]. All these arguments indicate reasonably that the barrier height of the closed gate decreases in the pathological environment of neuropathic pain. Hence, in the present study, we show the energy barrier *G* values at which the excitability changes such as membrane depolarization, ephaptic coupling, and spontaneous activity. The order of magnitude $10^{-20}$ J was used for the values of *G*, which is consistent with the order of magnitude for the values used in the experimental studies that used the unit kJ/mol = $0.17\times 10^{-20}$ J or kcal/mol = $0.69\times 10^{-20}$ J [30,31,32,33,34]. The second pathological event is the demyelination which is seen in several causes of neuropathic pain [60,61,62,63,64,65,66,67,68]. These two pathological events were applied within the quantum tunneling model to explain the three major pathophysiological mechanisms.

Furthermore, the gate length *L* varies according to the number of hydrophobic residues involved in forming the hydrophobic gate. For example, in [31,32] there are three hydrophobic residues and each pair is separated by another three residues; hence the whole path is composed of nine residues. From these references [31,32], it can be estimated that $L=(5-10)\times 10^{-10}$ m. However, voltage-gated channels are sealed off by one hydrophobic residue from each of the four subunits [23,24,25]. Therefore, by a simple comparison, if nine residue-paths yield $L=(5-10)\times 10^{-10}$ m, then it is expected that one residue-path yields $(0.55-1)\times 10^{-10}$ m. However, we included four setting values for the gate length *L* in our investigation, which are $L=0.5\times 10^{-10}$ m, $L=1\times 10^{-10}$ m, $L=1.5\times 10^{-10}$ m, and $L=2\times 10^{-10}$ m to cover wide range of possible values of *L*.

### 3.1. The Quantum Tunneling-Induced Membrane Depolarization and The Quantum Tunneling Currents

**Figure 8:**
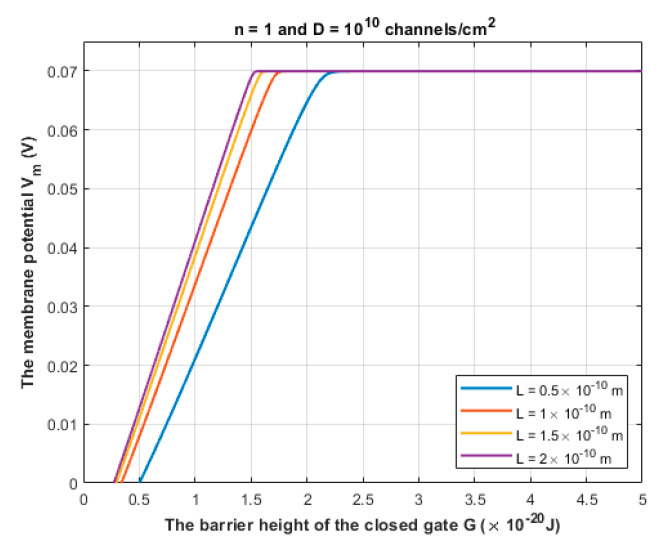
The figure represents the relationship between the membrane potential of neurons and the barrier height of the closed gate under the influence of the quantum tunneling of sodium ions through the closed gate of sodium channels. The relationship is investigated at different values of gate length *L* and according to the setting values above the figure.

**Figure 9:**
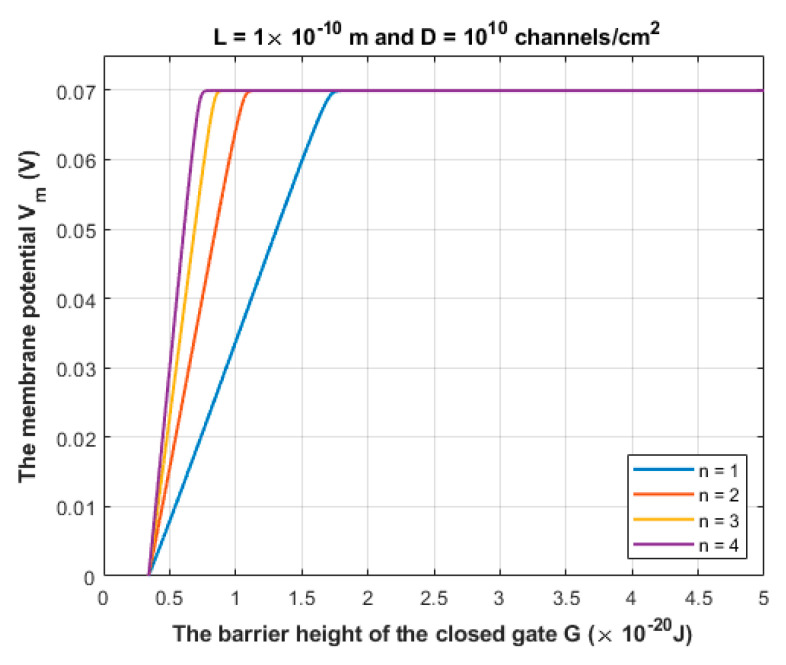
The figure represents the relationship between the membrane potential of neurons and the barrier height of the closed gate under the influence of the quantum tunneling of sodium ions. The relationship is investigated under different values of gate location *n* and according to the setting values above the figure.

According to Equation (10), sodium ions are expected to depolarize the membrane potential of the neurons via quantum tunneling through the closed channels. See Figure 8 and Figure 9.

**Figure 10:**
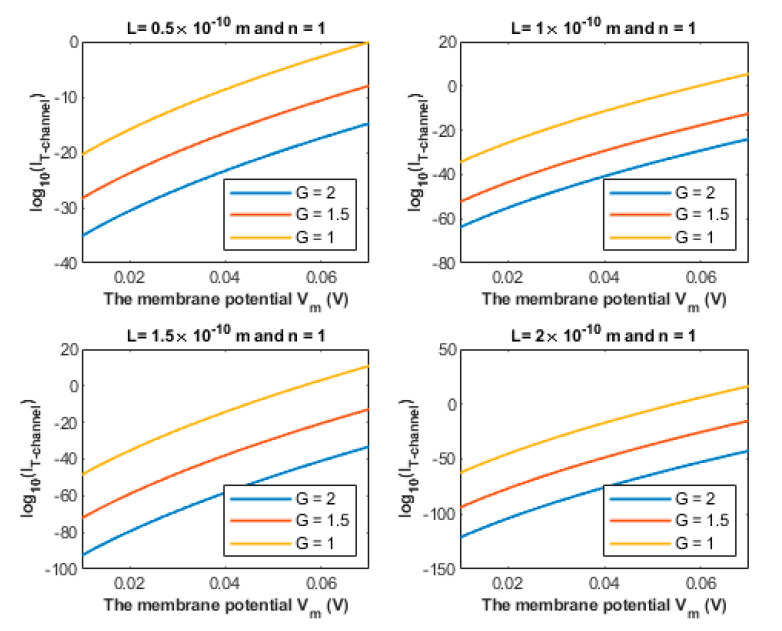
The figure represents the relationship between the common logarithm of the quantum tunneling current of sodium ions and the membrane potential of the neurons. The relationship is investigated at different values of G and according to the setting values above each figure. The unit of *G* in the legends is $10^{-20}$ J.

The quantum tunneling-induced depolarization is generated due to the net inward quantum tunneling current of sodium ions as presented mathematically in Equation (12). To explore how the membrane potential affects the inward tunneling current, it will be useful to investigate this relationship graphically. See Figure 10.

We chose the following values for *G*: $1\times 10^{-20}$ J, $1.5\times 10^{-20}$ J and $2\times 10^{-20}$ J in Figure 10 because the significant depolarization represented in Figure 8 and Figure 9 begins to occur at *G* values less than $2\times 10^{-20}$ J. Hence, focusing on these values is more reasonable.

### 3.2. The Quantum Ephaptic Coupling or ‘Quantum Synapse’

**Figure 11:**
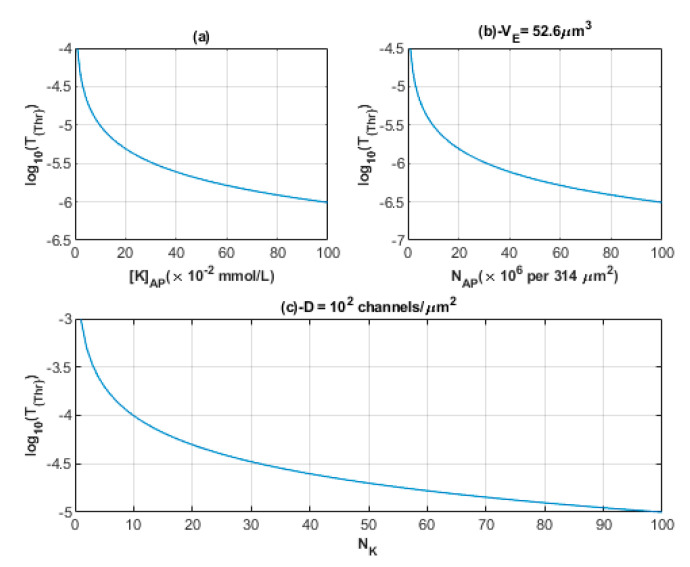
(**a**–**c**) The figures represent the relationship between the common logarithm of the quantum tunneling probability threshold and the change in potassium concentration during action potential, the number of potassium ions exit from 314 µm^2^ during an action potential, and the number of potassium ions that hit a single closed channel, respectively.

As we explained before, the quantum synapse is formed when the potassium ions of the minute concentration tunnel pass through the closed potassium channels in the membrane of adjacent neurons. These potassium ions can reach the threshold value of quantum tunneling probability that is required to induce an ectopic action potential. The relationship between the number of potassium ions and the threshold value of quantum tunneling probability is mathematically represented in Figure 11.

**Figure 12:**
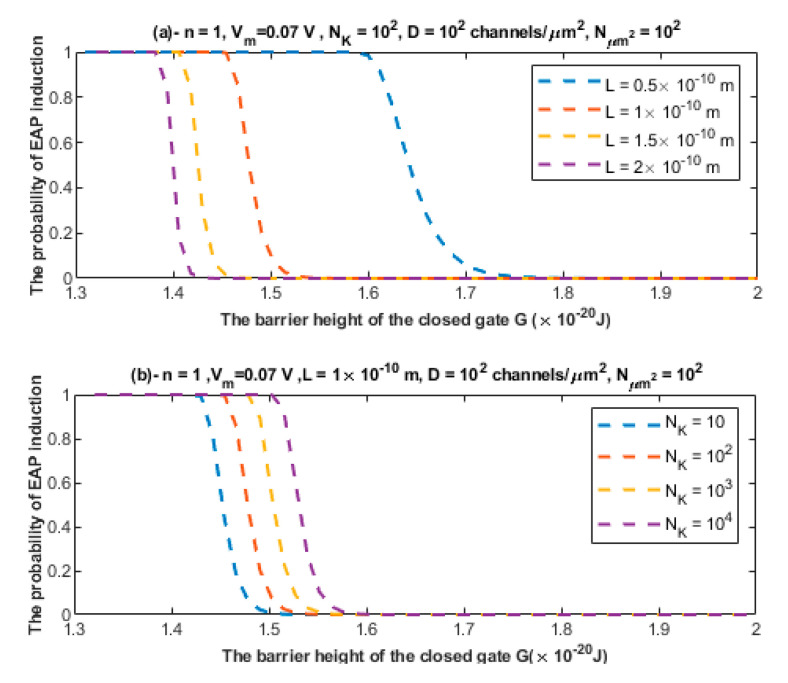
(**a**,**b**) The figure represents the relationship between the probability of inducing an ectopic action potential (EAP) and the barrier height of the closed gate *G* under the influence of different values of gate length *L* and different values of $N_{K}$, respectively. The relationships are investigated according to the setting values above each figure.

**Figure 13:**
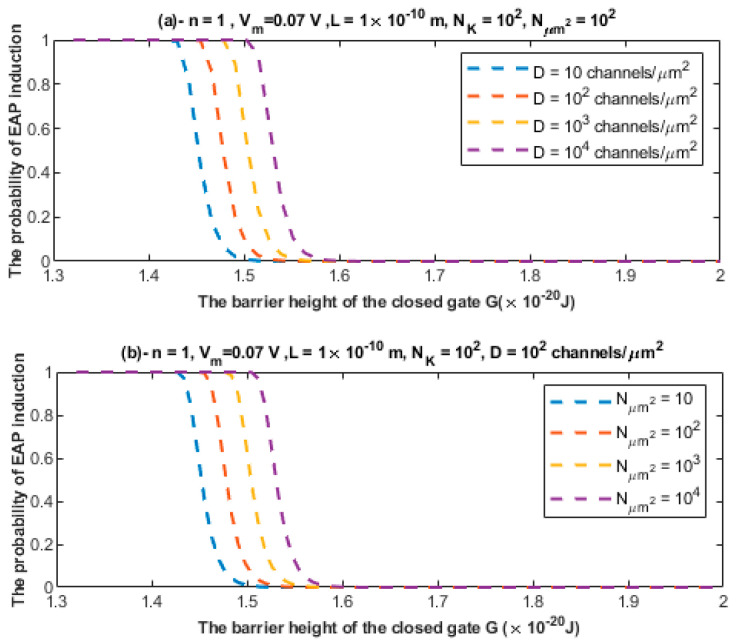
(**a**,**b**) The figure represents the relationship between the probability of inducing an ectopic action potential (EAP) and the barrier height of the closed gate *G* under the influence of different values of channels density *D* and different values of $N_{\mu m^{2}}$, respectively. The relationships are investigated according to the setting values above each figure.

According to the mathematical framework provided for the quantum synapse, it is clear that the quantum tunneling model predicts the probability of inducing an ectopic action potential. To assess the significance of such a probability, the relationship between the probability of inducing an ectopic action potential (EAP) and the barrier height of the closed channel is investigated at different setting values. See Figure 12 and Figure 13.

If a neuron is exposed to 10 stimulated neurons and each one carries 10 action potentials per second and assuming that the probability of EAP induction is 0.1, then by applying Equation (26), it is expected that the unstimulated neuron will transmit ectopic action potentials with a frequency of 10×10×0.1=10 ectopic action potentials per second.

### 3.3. The Quantum Tunneling-Induced Spontaneous Neuronal Firing

The quantum tunneling-induced spontaneous firing is based on the idea that the thermal environment can provide energy to the closed gate to reduce its barrier height without opening it. Such reduction can enhance the quantum tunneling of sodium ions and result in a spontaneous depolarization and spontaneous firing.

**Figure 14:**
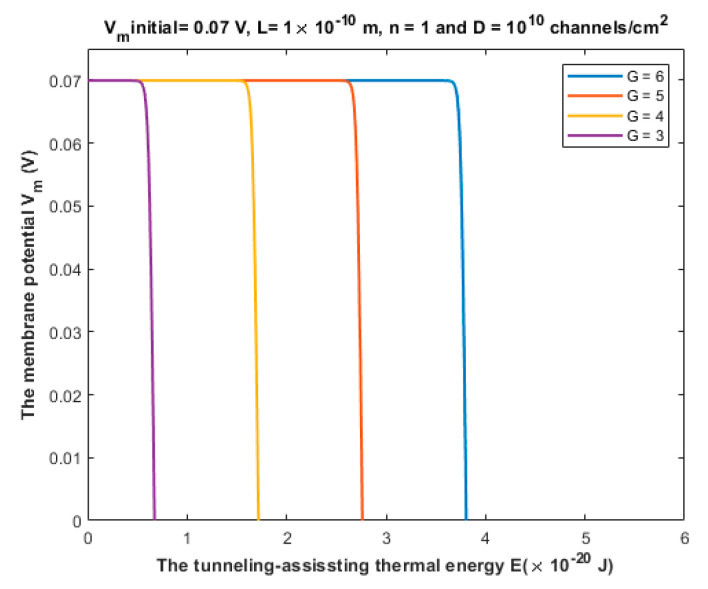
The figure represents the relationship between the membrane potential and the tunneling-assisted thermal energy *E* that lowers the barrier height of the closed gate, and thus augments the quantum tunneling. The relationship is investigated at different values of *G* and according to the setting values above the figure. The unit of *G* in the legend is $10^{-20}$ J.

Based on Equations (27)–(29), the quantum tunneling model predicts the ability of neurons to spontaneously fire and trigger ectopic action potentials via the thermally assisted quantum tunneling of sodium ions. To assess whether this mechanism can yield the generation of ectopic action potentials, see Figure 14.

The $V_{m(initial)}$ that is above Figure 14 represents the initial membrane potential that contributes to the kinetic energy of sodium ion *KE* when Equation (29) is applied to investigate the quantum tunneling-induced spontaneous firing.

**Figure 15:**
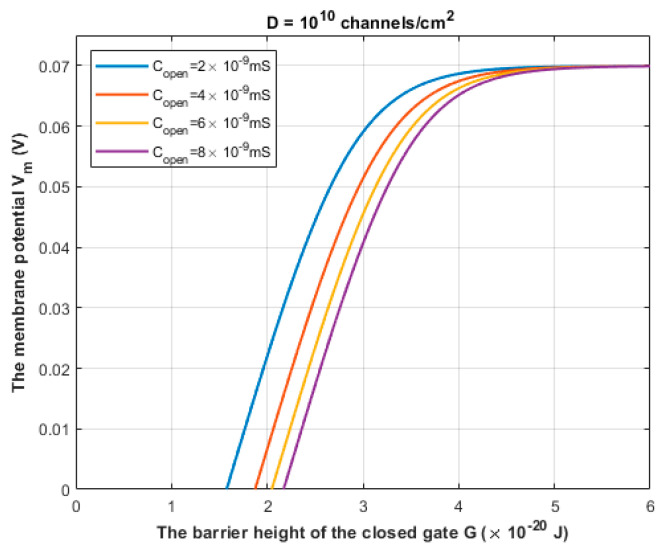
The figure represents the influence of the thermal opening of the voltage-gated sodium channels on the membrane potential at different values of open channel conductance.

Based on Equation (30), the relationship between the classical opening of sodium channels and the resting membrane potential can be evaluated to explore the effect of the pure thermal mechanism on the neuronal spontaneous firing. See Figure 15.

## 4. Discussion

### 4.1. Elaboration of The Ion Quantum Tunneling Model in The Context of Neuropathic Pain

The present study aimed to revisit the pathophysiological mechanisms of neuropathic pain in the context of quantum mechanics particularly by using the model of ions quantum tunneling. Three major aspects that will be reconsidered are: (1) the depolarized membrane potential of the neurons; (2) the ephaptic transmission between neurons; and (3) the spontaneous neuronal firing. The quantum tunneling model views the gate of voltage-gated channels as an energy barrier that blocks ion permeation. Moreover, it allows for ions to tunnel through the closed gate, which has a barrier height larger than the kinetic energy of the ion, via a quantum tunneling event. The results indicate that the extracellular cations such as sodium and potassium ions have higher quantum tunneling probability if they are compared with the intracellular cations. This is attributed to the higher kinetic energy of the extracellular cations. It has been shown previously that sodium and potassium ions were not able to affect the membrane potential at normal physiological parameters [16]. However, under certain pathological conditions, the quantum tunneling of ions is enhanced to the degree that it can change the membrane potential [17,18,19,20].

The quantum tunneling model predicts the ability of sodium ions to depolarize the membrane potential of neurons under the influence of the pathological effects of the neuropathic pain on the barrier height of the closed gate. When the barrier height of the closed gate drops, the quantum tunneling is augmented and is able to depolarize the membrane potential, which is indicated in Figure 8 and Figure 9. The degree of depolarization is modulated by the length of the gate *L* and the location of the gate *n*. The quantum tunneling-induced membrane depolarization is generated due to the net inward tunneling current of sodium ions as it represented in Figure 10. Moreover, the quantum tunneling current has unique features that make it distinctive from the classical current that is generated through an open channel. These features include: (1) The tunneling current does not require the opening of the closed gate to be generated. It requires the drop in the barrier height of the closed gate but without the full drop that makes the energy barrier of the gate less than the kinetic energy of the ion. (2) The tunneling current is continuous and persistent since it does not depend on the transient opening of the voltage-gated channels, which generates a transient current. (3) The tunneling current changes non-linearly with respect to the membrane voltage as represented in Figure 10. The quantum tunneling-induced membrane depolarization contributes to the hyperexcitability of the neurons and thus to the pathogenesis of neuropathic pain.

The second pathophysiological aspect in the neuropathic pain is the ephaptic transmission and the cross-talk between neurons. The ephaptic transmission has been observed experimentally and it has been implicated in the pathogenesis of neuropathic pain [10,11,12,13]. This type of transmission entails the ability of a stimulated neuron having an action potential to stimulate adjacent neurons without chemical or clear electric synapse. Therefore, its underlying mechanism seems elusive and not clearly defined. Moreover, the only possible mediator, according to the literature, of the ephaptic transmission is the endogenous electric field generated when the neuron fires [45,46]. However, these endogenous electric fields produce a small change in the membrane potential of the neighboring neurons by around 0.5 mV [45,46], which is a minute change relative to the membrane potential of the neuron of 70 mV. Therefore, the mechanism of the endogenous electric field can be only sufficient in changing the voltage of neurons and inducing an action potential in the case of highly packed cells with a small extracellular space as in the olfactory neurons [47]; otherwise no ephaptic transmission is expected to occur [47]. However, this type of transmission has been observed even with small changes in the membrane potential as we mentioned previously [45,47]. Hence, an alternative mechanism to ephaptic coupling is required to explain its process. The quantum tunneling model can provide a much more reasonable and consistent mechanism to explain the ephaptic coupling between neurons without the requirement of a strong endogenous electric field, which produces large changes in the membrane potential, and without even the requirement of large changes in potassium concentration. Therefore, the quantum interpretation is distinctive from the interpretations that depend on the significant changes in the chemical and electrical gradients. The quantum tunneling model views ephaptic coupling as a quantum synapse that is formed due the quantum tunneling of potassium ions that exit to the extracellular space between neurons as presented in Figure 5. These potassium ions tunnel through the closed potassium channels in the membrane of the neighboring unstimulated neurons to generate an inward tunneling current that can depolarize the membrane potential to the threshold needed to trigger an ectopic action potential. These potassium ions require a large number of trials to succeed in achieving a significant tunneling fraction sufficient to depolarize the membrane potential. Interestingly, the probability of inducing an ectopic action potential depends on the number of the closed potassium channels available for quantum tunneling of potassium ions, which increases if demyelination occurs because potassium channels become exposed [48,49,50,51]. Therefore, the quantum tunneling model predicts that the formation of quantum synapses is enhanced in demyelinated/unmyelinated neurons. This is consistent with the observations that indicated that ephaptic coupling, which corresponds to the quantum synapse in the present paper, was amplified if neurons are unmyelinated [47]. Therefore, the quantum synapse can be regarded as the underlying mechanism of ephaptic interactions. Additionally, the decrease in the barrier height of the closed gates of potassium channels strengthen the formation of quantum synapses because in this case the quantum tunneling of potassium ions is boosted. The results indicated that the quantum tunneling model can yield a significant probability of ectopic action potential induction according to Figure 12 and Figure 13.

**Figure 16:**
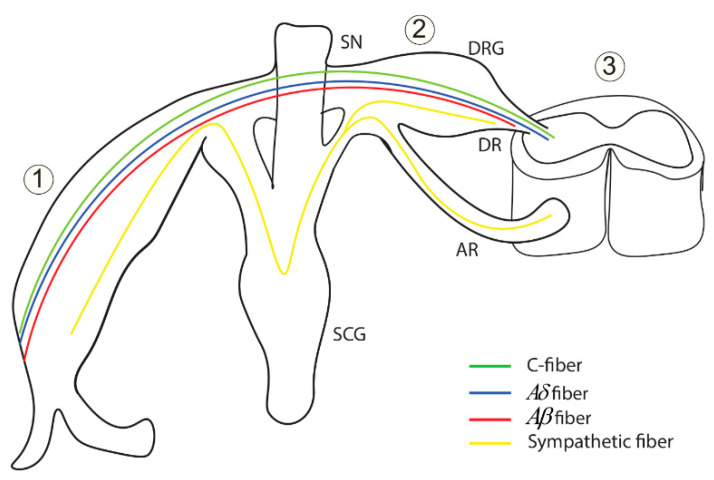
The figure represents the anatomical locations within the nervous system that provide the opportunity for the fibers to form the quantum synapses. 1: The anatomical location of the peripheral tract that includes the anterior and the posterior rami down to the targeted tissues. 2: The anatomical location that includes the spinal nerve (SN), the dorsal root ganglion (DRG), and the dorsal root (DR). 3: It represents the central nervous system, which includes the spinal cord segments and the brain (not shown). AR: anterior root; SCG: sympathetic chain ganglion.

The quantum synapses can be formed between the unmyelinated pain C-fibers themselves, between the unmyelinated pain C-fibers, and unmyelinated C-fibers of other sensations, which includes thermal and crude touch sensations, and between the unmyelinated C-fibers and the (de)myelinated pain Aδ fibers and the (de)myelinated Aβ fibers which transmits proprioception, vibrations, pressure, and discriminative touch especially when they are joined in the same peripheral tract before they diverge at the level of the dorsal horn of the spinal cord segment. Moreover, the quantum synapses can be formed between the unmyelinated C-fibers and the sympathetic fibers when they come close to each other at the level of dorsal root ganglion (DRG) due to the sprouting of the sympathetic fibers to the DRG that has been described in the literature [8,9,22] in addition to the other sites near the spinal nerve (SN) down to the peripheral tracts reaching the targeted tissues. See Figure 16.

Once the neuronal fibers diverge in separate tracts in the central nervous system (not shown in Figure 16 to avoid complexing and crowding the figure), the quantum synapses can also be formed but within the same tract because the tracts such as the spinothalamic pathway, which transmits pain, temperature, and crude touch, and the dorsal column–medial lemniscus pathway, which transmits proprioception, vibrations, pressure, and discriminative touch, become spatially separated to the degree that the minute changes in potassium concentration in one pathway cannot reach the other one.

The formation of quantum synapses explains the hyperexcitability among the C-pain fibers themselves because these synapses can induce an ectopic action potential in another neuron and thus a higher number of neurons and higher number of action potentials are transmitted to brain centers, which contributes to the generation of pain sensations. Moreover, the quantum synapses formation between pain fibers and non-pain fibers explains why mechanical stimuli such as touch or pressure (allodynia) and thermal stimuli (hyperalgesia) such as cold and warmth can trigger pain [1,7,8,22] because in this case the action potentials transmitted through non-pain fibers can be induced in the pain fibers via quantum tunneling of potassium ions through the closed channels. Furthermore, the quantum synapses can be formed between pain fibers and the sympathetic fibers since they can get the chance to become in close proximity to each other and this explains why sympathetic stimulation can trigger and maintain the neuropathic pain which is described as ‘sympathetically maintained pain’ [7,8,22]. When we say that the neurons are close to each other, we are not referring to the highly packed cells as in the olfactory pathway, rather we are referring to the neurons that have a large extracellular space that yields a small change in the membrane potential of the neurons and a small change in the potassium concentrations during action potential because in this case an alternative mechanism, other than the endogenous electric field, is required to explain the cross-talk between neurons. Accordingly, the quantum synapses can explain the cross-talk between the neurons with the same sensational modality and between the neurons with different sensational modalities.

The third pathophysiological aspect is the spontaneous firing and generation of ectopic action potential in the axons of injured neurons. The neurons can fire an action potential on their own once the membrane potential is depolarized to the threshold value, otherwise no action potential will be generated. According to Figure 14, it is clear that the thermally assisting energy provided from the thermal environment can lower the barrier height of the closed gate enhancing the tunneling probability of sodium ions. This enhancement leads to significant inward tunneling current of sodium ions that is sufficient to depolarize the neurons to the threshold value 55 mV. As the barrier height *G* decreases, the depolarization to the threshold value happens at lower values of thermally assisting energy *E*. This means that less thermal energy is required to induce the spontaneous firing. On the other hand, and according to Figure 15, the classical opening of closed voltage-gated channels can result in a spontaneous neuronal firing. However, there are unique features of the quantum tunneling-induced spontaneous firing that may make it the major factor in the generation of the ectopic action potentials. These features include: (1) According to the quantum tunneling model, the membrane potential changes with a higher rate with respect to the energy as is clear in Figure 14. This means that less energy is required to induce an ectopic action potential. On the other hand, the classical model predicts that the membrane potential changes with a lower rate with respect to the barrier height *G*, as presented in Figure 15. This means that more energy is required to induce an ectopic action potential according to the classical model. (2) According to the quantum tunneling model, it is not required to provide a thermal energy *E* equivalent or higher than the barrier height *G* to induce an ectopic action potential. For example, in Figure 14, when $G=3\times 10^{-20}$ J, a thermal energy *E* that is less than $1\times 10^{-20}$ J is required to depolarize the membrane potential to the threshold of 55 mV and thus to induce an ectopic action potential. On the other hand, the full energy of the barrier height *G* must be provided to depolarize the membrane potential significantly and induce an ectopic action potential as is clearly represented in Figure 15. Therefore, it is more energetically favorable for the neurons to spontaneously fire quantum mechanically rather than classically. The quantum tunneling-induced spontaneous firing, which is energetically favorable, contributes to the hyperexcitability and the ongoing neuronal activity which explains the ongoing pain in patients with neuropathic pain [1,7,8,22].

### 4.2. The Potential Applicability of The Quantum Tunneling Model in The Context of Neuropathic Pain

The applicability of the quantum tunneling model can be demonstrated in two sections:

1. Pathophysiological implications: There are certain unique features in the quantum tunneling model that make it distinctive from any classical model. These features can be deduced from Equation (2) and include: (1) The exponential dependence on the mass of the ion and the length of the gate to determine the quantum tunneling probability and quantum conductance. This would serve to be a promising strategy to test the validity of the role of the quantum behavior of ions. For example, as sodium and potassium ions have different masses, then observing an exponential difference between ions in terms of tunneling probability and quantum conductance is expected. Interestingly, the exponential mass difference can be also applied on other ions such as lithium and hydrogen ions. Similarly, observing an exponential dependence on the length of the gate will add additional supporting evidence. (2) Another implication that indicates strongly to the quantum tunneling behavior of ions is observing a depolarization action by potassium ions especially when there is a decrease in the energy barrier of the closed gate. Classically, when there is a gain-of-function mutation in potassium channels or when these channels open, it is expected that the outward potassium current will occur, which tends to hyperpolarize the membrane potential. However, according to the quantum tunneling model, it is expected that the inward potassium current will occur, which tends to depolarize the membrane potential.
2. Pharmacological implications: These implications are crucial to be demonstrated to exhibit the beneficial consequences of the quantum tunneling model. If the pathophysiological implications can be tested experimentally and the quantum coherence of ions can be proven in ion channels, especially in the narrow hydrophobic gate, then we can propose this class of medications, which is ‘quantum decoherence inducers’ or ‘quantum coherence destroyers’ or ‘quantum decoherence agents’. All of these coined terms can be used to describe the ability of these proposed drugs to collapse the quantum wave or to weaken the quantum behavior of ions. If this quantum decoherence happens, then all the proposed pathophysiological mechanisms will be diminished or will be eliminated. Consequently, these proposed drugs can contribute significantly to pain relief and achieve satisfactory clinical outcomes. Our proposal for these drugs requires further investigation and an interdisciplinary cooperation to test the potential applicability of this proposal. We propose these drugs to attract the attention of all researchers across the different related disciplines to the possible applicability of the quantum tunneling model to act actively in the treatment of the neuropathic pain or even in the prevention of neuropathic pain by implementing our understanding of the pathophysiological mechanisms from the quantum mechanical perspective.

### 4.3. Limitations

As our study belongs to the field of quantum biology, there are the concerns about the quantum decoherence issues due to the noisy and hot biological environment. However, these concerns no longer represent a serious barrier to applying the principles of quantum mechanics due to the accumulating theoretical and experimental evidence that indicates strongly that the quantum coherence can be maintained in the biological environment [69,70,71]. Additionally, our quantum tunneling model is applied on ions, which are not the typical particles that quantum mechanics is applied to due to their larger mass if it is compared with the mass of protons and electrons. However, recent experimental studies showed that the quantum behavior can be evident on larger mass scale than the mass scale of ions, which includes the mass scale of peptides and molecules composed of several hundreds of atoms and with a mass up to 6000 AMU [72,73], which is much larger than sodium and potassium ions. Furthermore, as the barrier shape of the closed gate remains to be further specified, the symmetrical Eckert potential is required to show its reasonable experimental validity to provide sensible estimation of the tunneling probability and the quantum conductance of ion channels.

## 5. Conclusions

**Figure 17:**
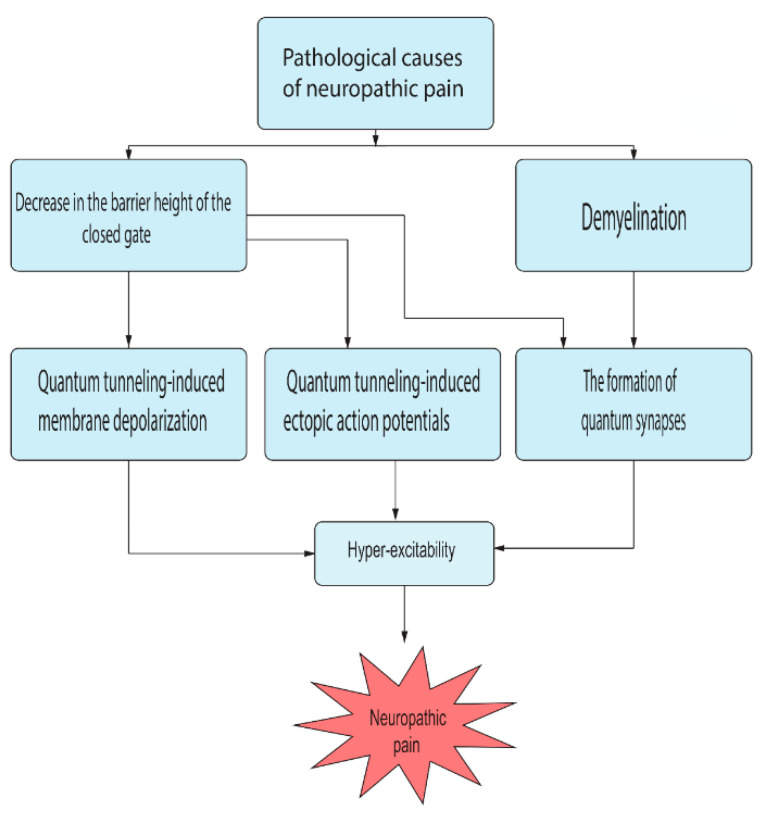
A schematic diagram of the quantum aspects in the pathophysiology of neuropathic pain.

The pathological causes of neuropathic pain can decrease the barrier height of the closed gate of voltage-gated channels and induce demyelination. These two major pathological effects contribute to the emergence of obvious quantum behavior of sodium and potassium ions. Three pathophysiological aspects can be revisited in the context of the quantum tunneling model, which contribute significantly to the pathogenesis of neuropathic pain. The first aspect is the quantum tunneling-induced membrane depolarization which is mediated by the inward quantum tunneling current of sodium ions. The second aspect is the formation of quantum synapses which are formed by the quantum tunneling of potassium ions that exit during action potentials. These quantum synapses explain the ephaptic interactions between injured neurons. The third aspect is the quantum tunneling-induced spontaneous neuronal firing and generation of ectopic action potentials. All these three pathophysiological aspects create a state of hyperexcitability among pain fibers that explains the clinical manifestations in the patients with neuropathic pain. See Figure 17.

## Data Availability

Data available upon request from the corresponding author.

## References

[1] Colloca L., Ludman T., Bouhassira D., Baron R., Dickenson A.H., Yarnitsky D., Freeman R., Truini A., Attal N., Finnerup N.B. (2017). Neuropathic pain. Nat. Rev. Dis. Primers.

[2] Sapunar D., Ljubkovic M., Lirk P., McCallum J.B., Hogan Q.H. (2005). Distinct Membrane Effects of Spinal Nerve Ligation on Injured and Adjacent Dorsal Root Ganglion Neurons in Rats. Anesthesiology.

[3] Lee Y., Lee C.H., Oh U. (2005). Painful channels in sensory neurons. Mol. Cells.

[4] Wu B., Su X., Zhang W., Zhang Y.-H., Feng X., Ji Y.-H., Tan Z.-Y. (2021). Oxaliplatin Depolarizes the IB4– Dorsal Root Ganglion Neurons to Drive the Development of Neuropathic Pain through TRPM8 in Mice. Front. Mol. Neurosci..

[5] Coggan J.S., Ocker G.K., Sejnowski T.J., Prescott S.A. (2011). Explaining pathological changes in axonal excitability through dynamical analysis of conductance-based models. J. Neural Eng..

[6] Xie W., Strong J., Meij J., Zhang J.-M., Yu L. (2005). Neuropathic pain: Early spontaneous afferent activity is the trigger. Pain.

[7] Djouhri L., Koutsikou S., Fang X., McMullan S., Lawson S.N. (2006). Spontaneous Pain, Both Neuropathic and Inflammatory, Is Related to Frequency of Spontaneous Firing in Intact C-Fiber Nociceptors. J. Neurosci..

[8] Campbell J.N., Meyer R.A. (2006). Mechanisms of neuropathic pain. Neuron.

[9] Nickel F.T., Seifert F., Lanz S., Maihöfner C. (2012). Mechanisms of neuropathic pain. Eur. Neuropsychopharmacol..

[10] Seltzer Z., Devor M. (1979). Ephaptic transmission in chronically damaged peripheral nerves. Neurology.

[11] Sorkin L.S., Eddinger K.A., Woller S.A., Yaksh T.L. (2018). Origins of antidromic activity in sensory afferent fibers and neurogenic inflammation. Seminars in Immunopathology.

[12] Asmedi A., Wibowo S., Meliala L. (2018). Ephaptic crosstalk in Painful Diabetic Neuropathy: An electrodiagnostic study. J. Med Sci. (Berk. Ilmu Kedokt.).

[13] Gould H.J., Soignier R.D., Cho S.R., Hernandez C., Diamond I., Taylor B.K., Paul D. (2014). Ranolazine attenuates mechanical allodynia associated with demyelination injury. Pain Med..

[14] Kim Y., Bertagna F., D’Souza E.M., Heyes D.J., Johannissen L.O., Nery E.T., Pantelias A., Jimenez A.S.-P., Slocombe L., Spencer M.G. (2021). Quantum Biology: An Update and Perspective. Quantum Rep..

[15] Calvillo L., Redaelli V., Ludwig N., Qaswal A.B., Ghidoni A., Faini A., Rosa D., Lombardi C., Pengo M., Bossolasco P. (2022). Quantum Biology Research Meets Pathophysiology and Therapeutic Mechanisms: A Biomedical Perspective. Quantum Rep..

[16] Barjas Qaswal A. (2019). Quantum Tunneling of Ions through the Closed Voltage-Gated Channels of the Biological Membrane: A Mathematical Model and Implications. Quantum Rep..

[17] Qaswal A.B., Ababneh O., Khreesha L., Al-Ani A., Suleihat A., Abbad M. (2021). Mathematical Modeling of Ion Quantum Tunneling Reveals Novel Properties of Voltage-Gated Channels and Quantum Aspects of Their Pathophysiology in Excitability-Related Disorders. Pathophysiology.

[18] Qaswal A. (2019). A Theoretical Study to Explain the Referred Pain Phenomenon and Its Characteristics via Quantum Tunneling of Potassium Ions through the Channels of Neuronal Membrane. NeuroQuantology.

[19] Qaswal A.B. (2019). The Myelin Sheath Maintains the Spatiotemporal Fidelity of Action Potentials by Eliminating the Effect of Quantum Tunneling of Potassium Ions through the Closed Channels of the Neuronal Membrane. Quantum Rep..

[20] Alrabayah M., Qaswal A.B., Suleiman A., Khreesha L. (2020). Role of Potassium Ions Quantum Tunneling in the Pathophysiology of Phantom Limb Pain. Brain Sci..

[21] Balzani E., Fanelli A., Malafoglia V., Tenti M., Ilari S., Corraro A., Muscoli C., Raffaeli W. (2021). A Review of the Clinical and Therapeutic Implications of Neuropathic Pain. Biomedicines.

[22] Bridges D., Thompson S.W., Rice A.S. (2001). Mechanisms of neuropathic pain. Br. J. Anaesth..

[23] Aryal P., Sansom M.S., Tucker S.J. (2014). Hydrophobic Gating in Ion Channels. J. Mol. Biol..

[24] Oelstrom K., Goldschen-Ohm M.P., Holmgren M., Chanda B. (2014). Evolutionarily conserved intracellular gate of voltage-dependent sodium channels. Nat. Commun..

[25] Labro A.J., Snyders D.J. (2012). Being Flexible: The Voltage-Controllable Activation Gate of Kv Channels. Front. Pharmacol..

[26] Hering S., Zangerl-Plessl E.M., Beyl S., Hohaus A., Andranovits S., Timin E.N. (2018). Calcium channel gating. Pflügers Arch.-Eur. J. Physiol..

[27] Chandra A.K. (1994). Introductory Quantum Chemistry.

[28] Eckart C. (1930). The Penetration of a Potential Barrier by Electrons. Phys. Rev..

[29] Miyazaki T. (2004). Atom Tunneling Phenomena in Physics, Chemistry and Biology.

[30] Rao S., Klesse G., Stansfeld P.J., Tucker S.J., Sansom M.S.P. (2019). A heuristic derived from analysis of the ion channel structural proteome permits the rapid identification of hydrophobic gates. Proc. Natl. Acad. Sci. USA.

[31] Rao S., Lynch C.I., Klesse G., Oakley G.E., Stansfeld P.J., Tucker S.J., Sansom M.S.P. (2018). Water and hydrophobic gates in ion channels and nanopores. Faraday Discuss..

[32] Rao S., Klesse G., Lynch C.I., Tucker S.J., Sansom M.S.P. (2021). Molecular Simulations of Hydrophobic Gating of Pentameric Ligand Gated Ion Channels: Insights into Water and Ions. J. Phys. Chem. B.

[33] Tepper H.L., Voth G.A. (2006). Mechanisms of Passive Ion Permeation through Lipid Bilayers: Insights from Simulations. J. Phys. Chem. B.

[34] Khavrutskii I.V., Gorfe A.A., Lu B., McCammon J.A. (2009). Free Energy for the Permeation of Na^+^ and Cl^−^ Ions and Their Ion-Pair through a Zwitterionic Dimyristoyl Phosphatidylcholine Lipid Bilayer by Umbrella Integration with Harmonic Fourier Beads. J. Am. Chem. Soc..

[35] Al-Rawashdeh B.M., Qaswal A.B., Suleiman A., Zayed F.M., Al-Rawashdeh S.M., Tawalbeh M., Khreesha L., Alzubaidi A., Al-Zubidi E., Ghala Z. (2022). The Quantum Tunneling of Ions Model Can Explain the Pathophysiology of Tinnitus. Brain Sci..

[36] Payandeh J., El-Din T.G., Scheuer T., Zheng N., Catterall W.A. (2012). Crystal structure of a voltage-gated sodium channel in two potentially inactivated states. Nature.

[37] Cuello L.G., Jogini V., Cortes D.M., Perozo E. (2010). Structural mechanism of C-type inactivation in K+ channels. Nature.

[38] Bagnéris C., Naylor C.E., McCusker E.C., Wallace B.A. (2014). Structural model of the open–closed–inactivated cycle of prokaryotic voltage-gated sodium channels. J. Gen. Physiol..

[39] Serway R.A., Moses C.J., Moyer C.A. (2004). Modern Physics.

[40] Chen F., Hihath J., Huang Z., Li X., Tao N. (2007). Measurement of Single-Molecule Conductance. Annu. Rev. Phys. Chem..

[41] Tsymbal E.Y., Mryasov O.N., LeClair P.R. (2003). Spin-dependent tunnelling in magnetic tunnel junctions. J. Phys. Condens. Matter.

[42] Bertil H., Bertil H. (2001). Ion Channels of Excitable Membranes.

[43] Hall J.E., Hall M.E. (2020). Guyton and Hall Textbook of Medical Physiology E-Book.

[44] Qaswal A.B. (2020). Quantum Electrochemical Equilibrium: Quantum Version of the Goldman–Hodgkin–Katz Equation. Quantum Rep..

[45] Anastassiou C.A., Perin R., Markram H., Koch C. (2011). Ephaptic coupling of cortical neurons. Nat. Neurosci..

[46] Anastassiou C.A., Koch C. (2015). Ephaptic coupling to endogenous electric field activity: Why bother?. Curr. Opin. Neurobiol..

[47] Bokil H., Laaris N., Blinder K., Ennis M., Keller A. (2001). Ephaptic Interactions in the Mammalian Olfactory System. J. Neurosci..

[48] Ouyang H., Sun W., Fu Y., Li J., Cheng J.-X., Nauman E., Shi R. (2010). Compression Induces Acute Demyelination and Potassium Channel Exposure in Spinal Cord. J. Neurotrauma.

[49] Waxman S.G. (1982). Membranes, Myelin, and the Pathophysiology of Multiple Sclerosis. New Engl. J. Med..

[50] Jukkola P., Lovett-Racke A.E., Zamvil S.S., Gu C. (2012). K+ channel alterations in the progression of experimental autoimmune encephalomyelitis. Neurobiol. Dis..

[51] Shi R., Sun W. (2011). Potassium channel blockers as an effective treatment to restore impulse conduction in injured axons. Neurosci. Bull..

[52] Buonocore M., Bonezzi C., Barolat G. (2008). Neurophysiological Evidence of Antidromic Activation of Large Myelinated Fibres in Lower Limbs During Spinal Cord Stimulation. Spine.

[53] Hamada M.S., Kole M.H.P. (2015). Myelin Loss and Axonal Ion Channel Adaptations Associated with Gray Matter Neuronal Hyperexcitability. J. Neurosci..

[54] Taniguchi M., Yamada Y., Fukumoto Y., Sawano S., Minami S., Ikezoe T., Watanabe Y., Kimura M., Ichihashi N. (2017). Increase in echo intensity and extracellular-to-intracellular water ratio is independently associated with muscle weakness in elderly women. Eur. J. Appl. Physiol..

[55] Morris C.E. (2011). Voltage-Gated Channel Mechanosensitivity: Fact or Friction?. Front. Physiol..

[56] Wang J.A., Lin W., Morris T., Banderali U., Juranka P.F., Morris C.E. (2009). Membrane trauma and Na^+^ leak from Nav1. 6 channels. Am. J. Physiol.-Cell Physiol..

[57] Beyder A., Rae J.L., Bernard C., Strege P.R., Sachs F., Farrugia G. (2010). Mechanosensitivity of Nav1.5, a voltage-sensitive sodium channel. J. Physiol..

[58] Liu L., Yang T.M., Liedtke W., Simon S.A. (2006). Chronic IL-1β Signaling Potentiates Voltage-Dependent Sodium Currents in Trigeminal Nociceptive Neurons. J. Neurophysiol..

[59] Binshtok A.M., Wang H., Zimmermann K., Amaya F., Vardeh D., Shi L., Brenner G.J., Ji R.R., Bean B.P., Woolf C.J. (2008). Nociceptors are interleukin-1β sensors. J. Neurosci..

[60] Gillespie C., Sherman D.L., Fleetwood-Walker S.M., Cottrell D.F., Tait S., Garry E.M., Wallace V.C., Ure J., Griffiths I.R., Smith A. (2000). Peripheral Demyelination and Neuropathic Pain Behavior in Periaxin-Deficient Mice. Neuron.

[61] Khan N., Smith M.T. (2014). Multiple sclerosis-induced neuropathic pain: Pharmacological management and pathophysiological insights from rodent EAE models. Inflammopharmacology.

[62] Waxman S.G. (1989). Demyelination in spinal cord injury. J. Neurol. Sci..

[63] Totoiu M.O., Keirstead H.S. (2005). Spinal cord injury is accompanied by chronic progressive demyelination. J. Comp. Neurol..

[64] Stadelmann C., Wegner C., Brück W. (2011). Inflammation, demyelination, and degeneration—recent insights from MS pathology. Biochim. Et Biophys. Acta (BBA)-Mol. Basis Dis..

[65] Cerda F.P., Gomez M.V.S., Matute C. (2016). The link of inflammation and neurodegeneration in progressive multiple sclerosis. Mult. Scler. Demyelinating Disord..

[66] Kelley R.E. (2006). Ischemic demyelination. Neurol. Res..

[67] Sharma K.R., Cross J., Farronay O., Ayyar D.R., Shebert R.T., Bradley W.G. (2002). Demyelinating Neuropathy in Diabetes Mellitus. Arch. Neurol..

[68] Bril V., Blanchette C.M., Noone J.M., Runken M.C., Gelinas D., Russell J.W. (2016). The dilemma of diabetes in chronic inflammatory demyelinating polyneuropathy. J. Diabetes Its Complicat..

[69] Kong J., Jiménez-Martínez R., Troullinou C., Lucivero V.G., Tóth G., Mitchell M.W. (2020). Measurement-induced, spatially-extended entanglement in a hot, strongly-interacting atomic system. Nat. Commun..

[70] Salari V., Tuszynski J., Rahnama M., Bernroider G. (2011). Plausibility of quantum coherent states in biological systems. J. Phys. Conf. Ser..

[71] Hagan S., Hameroff S.R., Tuszyński J.A. (2002). Quantum computation in brain microtubules: Decoherence and biological feasibility. Phys. Rev. E.

[72] Gerlich S., Eibenberger S., Tomandl M., Nimmrichter S., Hornberger K., Fagan P.J., Tüxen J., Mayor M., Arndt M. (2011). Quantum interference of large organic molecules. Nat. Commun..

[73] Shayeghi A., Rieser P., Richter G., Sezer U., Rodewald J.H., Geyer P., Martinez T.J., Arndt M. (2020). Matter-wave interference of a native polypeptide. Nat. Commun..

